# Prediction and Prevention of Intraoperative Hypotension with the Hypotension Prediction Index: A Narrative Review

**DOI:** 10.3390/jcm11195551

**Published:** 2022-09-22

**Authors:** Tatiana Sidiropoulou, Marina Tsoumpa, Panayota Griva, Vasiliki Galarioti, Paraskevi Matsota

**Affiliations:** Second Department of Anesthesiology, Attikon University Hospital, National and Kapodistrian University of Athens, 12462 Athens, Greece

**Keywords:** intraoperative hypotension, machine learning, hypotension prediction index

## Abstract

Intraoperative hypotension is common and has been associated with adverse events. Although association does not imply causation, predicting and preventing hypotension may improve postoperative outcomes. This review summarizes current evidence on the development and validation of an artificial intelligence predictive algorithm, the Hypotension Prediction (HPI) (formerly known as the Hypotension Probability Indicator). This machine learning model can arguably predict hypotension up to 15 min before its occurrence. Several validation studies, retrospective cohorts, as well as a few prospective randomized trials, have been published in the last years, reporting promising results. Larger trials are needed to definitively assess the usefulness of this algorithm in optimizing postoperative outcomes.

## 1. Introduction

“It is better and more useful to meet a problem in time than to seek a remedy after the damage is done” is a Latin saying of the mid-13th century, according to the Oxford Dictionary of Phrase and Fable, but it can also be applied in contemporary preventive medicine. In fact, artificial intelligence prediction models using machine learning techniques are increasingly developed and used in various clinical settings, including anesthesiology [1]. In this regard, the ability to predict future (adverse) events and postoperative morbidity and mortality on the basis of preoperative or intraoperative data has long been sought [2,3,4,5,6]. 

Intraoperative hypotension (IOH) is common during surgical procedures. It may be caused by anesthesia drugs, underlying comorbidities of the patient, or by the surgical procedure per se [7]. Given that IOH has been associated with increased postoperative myocardial injury, acute kidney injury, and mortality in the postoperative period [8,9,10,11,12], preventing IOH may potentially decrease the occurrence of these adverse events.

In this narrative review, we summarize current evidence on the use of a newly implemented machine learning algorithm, the Hypotension Prediction Index (HPI) [13,14], developed by Edwards Lifesciences (Irvine, CA, USA). The hypothetical benefit of this prediction model would be to provide information about an imminent episode of hemodynamic instability and hypotension as well as additional data on its underlying cause. 

## 2. Methodology and Study Selection

The electronic databases MEDLINE, the Cochrane Central Register of Controlled Clinical Trials, Science Direct, and Scopus were searched using the following terms: (“Intraoperative” OR “Postoperative”) AND (“Hypotension” OR “Arterial Pressure”) AND (“Prediction” OR “Prevention” OR “Probability” OR “Machine-learning” OR “Algorithm”) AND (“Index” OR ‘Indicator’). The research was limited by language (English only) and publication date (from 1 January 2015 to 12 June 2022). Lastly, bibliographies of retrieved articles were scrutinized for any relevant trials not yet identified in the primary search. We excluded studies that did not refer to IOH prediction via the Hypotension Prediction Index.

We sought randomized controlled trials, prospective or retrospective cohort studies, as well as systematic or narrative reviews, referring to the use of the HPI (Edwards Lifesciences, Irvine, CA, USA). We excluded studies or publications that did not include the use of this Index. The search results retrieved more than 30 publications concerning the use of the Hypotension Prediction Index. 

## 3. Clinical Importance of Intraoperative Hypotension 

Intraoperative hypotension occurs frequently during surgery, and its prevalence varies according to the stated definition [15]. Multiple causes of hypotension during anesthesia have been identified, leading to a subclassification of IOH in postinduction hypotension, early intraoperative hypotension, and late intraoperative hypotension based on the different factors that influence blood pressure intraoperatively [16]. Regardless of the cause of IOH, the risk of adverse events seems not to be influenced by its timing but rather by the severity and the duration of IOH [17,18]. Recently a prospective randomized trial on high-risk patients undergoing major surgery was published [11]. The authors reported a significant reduction in postoperative organ dysfunction in the group where blood pressure was maintained within the 10% range from the baseline preinduction values. This finding, along with a plethora of retrospective associative studies between IOH and adverse postoperative outcomes, prompted the recommendation to maintain intraoperative mean arterial pressure (MAP) above 60–70 mmHg [19]. Values below this threshold are associated with myocardial injury, acute kidney injury, and death. Similarly, systolic arterial pressures below 100 mm Hg are associated with myocardial injury and death [19]. 

## 4. Rationale and Development of the Hypotension Prediction Index

Treatment of hypotension is currently reactive, which means that it starts after a hypotensive effect occurs. The type of treatment will depend on various hemodynamic variables that can be provided by basic or advanced monitoring techniques. However, even if these techniques can supply detailed knowledge on the actual hemodynamic status of the patient, they cannot predict future hypotensive events. Therefore, hypotension will occur, and given that even brief episodes of IOH can prove to be deleterious for the patient, the need for a prediction model for IOH becomes apparent.

The HPI prediction model was developed by Hatib et al. [13] with the help of machine learning. The HPI can predict a hypotensive event, defined as MAP ≤ 65 mmHg, for more than 1 min, 5 to 15 min before it occurs. The HPI is an algorithm in which the 23 features of the arterial waveform with the best predictive values are incorporated out of a possible combination of more than 2.6 million features [13]. It produces a number ranging from 0 to 100 (from no to certain hypotensive event), wherein a value of 85 is considered the threshold to initiate treatment [13]. These values are obtained by an arterial cannula connected to a commercially available Acumen IQ transducer (Edwards Lifesciences, Irvine, CA, USA) that analyzes signals from the arterial waveform and transfers them to the Hemosphere monitor. Recently, a noninvasive method using the arterial waveform of a finger cuff and the volume clamp method (ClearSight, Edwards Lifesciences, Irvine, CA, USA), described by Peñáz et al. [20], as well as the Physiocal calibration by Wesseling accounting for changes in unloaded volume [21], has also been made commercially available [22,23,24,25]. 

The Hypotension Prediction Index was validated both internally and externally in surgical and intensive care unit (ICU) patients [13,14]. The algorithm predicted arterial hypotension with high sensitivity and specificity and an area under the receiver operating characteristic curve (AUC) of 0.95 to 0.97 for 15 to 5 min before a hypotensive event [14]. Therefore, with the use of the HPI algorithm, hypotension can be theoretically predicted and subsequently prevented with adequate treatment. 

## 5. Clinical Guidance and Intervention with the Hypotension Prediction Index 

**Figure 1:**
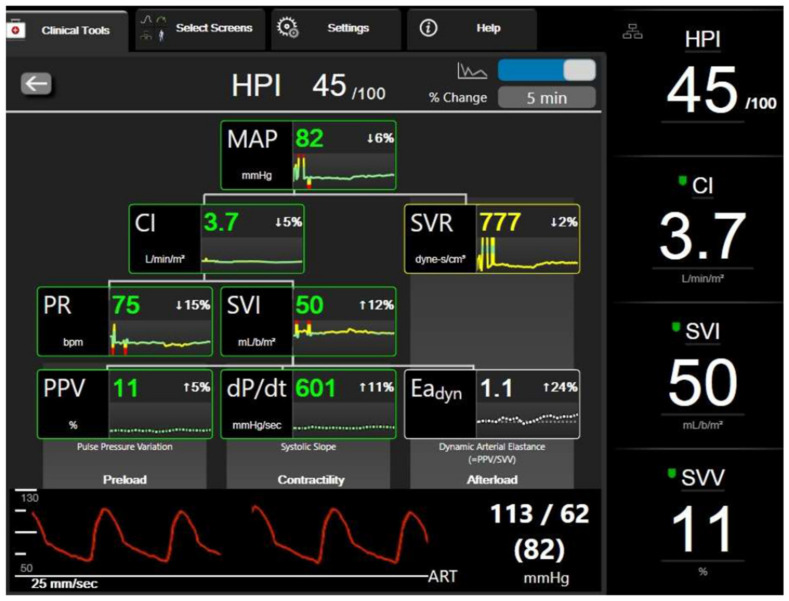
The secondary screen of the HPI algorithm (Edwards Lifesciences, Irvine, CA, USA).

Along with the HPI algorithm incorporated in the Hemosphere monitor, there is the option of a secondary screen (Figure 1) which provides valuable information on the possible cause of the future hypotensive event [26]. The secondary screen displays several hemodynamic variables in a decision tree manner and is divided into three areas which represent three leaf nodes: (i) preload, indicated by the stroke volume variation (SVV), (ii) contractility of the heart, specified by the dP/dt_MAX_ derived from the arterial waveform analysis, and (iii) afterload which is represented by the dynamic arterial elastance (Eadyn). Arterial dP/dt_MAX_ has been shown to correlate well with LV dP/dt_MAX_, measured by transthoracic echocardiography, in critically ill patients [27,28]. Eadyn, defined as the ratio between pulse pressure variation (PPV) and SVV, has been proposed as an index to predict the arterial blood pressure response to a fluid challenge in preload-dependent patients [29]. It is, therefore, a dynamic index that encompasses the interaction between arterial pressure and stroke volume during a respiratory cycle and can be interpreted as a measure of arterial load [30].

When a hypotensive episode is imminent, and the HPI value is above 80–85%, a clinician can obtain significant hemodynamic data from the secondary screen, guiding the treatment to the underlying cause of hypotension. Fluids, inotropes, or vasopressors can be administered for SVV, dP/dt_MAX_, or Eadyn significant changes, respectively. Hence the HPI algorithm might also be described as an index of hemodynamic instability. A rapidly increasing value of HPI will denote hemodynamic changes in the patient, which will ultimately alert the clinician to act proactively before the hypotension becomes overt.

## 6. Clinical Application of the Hypotension Prediction Index

The HPI algorithm has been clinically validated in the operating room but also in ICU patients (Table 1 and Table 2). For a more comprehensive exposition, they are presented in subgroups that are based on the study population and the technique applied. 

### 6.1. Invasive Arterial Waveform Analysis

#### 6.1.1. General Noncardiac Surgery

The performance of the HPI algorithm has been validated clinically in various studies of patients undergoing noncardiac [14,22,23,24,25,26,38] or cardiac [31,37] surgery. In the original study describing the derivation and validation cohorts of the algorithm development, Hatib et al. [13] used data from 554 surgical and ICU patients for the internal and external validation of the algorithm. They reported a sensitivity and specificity of 88% and 87% 15 min before a hypotensive event (area under the curve (AUC) 0.95); 89% and 90% 10 min before (AUC 0.95); 92% and 92% 5 min before (AUC, 0.97). In their cohort, the investigators excluded hypotensive events caused by the clinical intervention (e.g., vascular clamping, patient positioning) and arbitrarily used a binary definition of hypotension (hypotensive events defined as MAP < 65 mmHg and nonhypotensive events defined as MAP > 75 mmHg) leaving a gray zone in between. A recent analysis of the HPI algorithm underlined the problems generated by this selection bias [41]. In fact, Enevoldsen and Vistisen [41] analyzed data from the original [13] as well as subsequent validation studies and found that the AUC of all studies was skewed towards high specificity. This, as explained by the authors and an accompanying editorial [34], could potentially lead to an overestimation of the risk of hypotension with resulting overtreatment. On the other hand, the risk of hypertension has not emerged from the majority of the HPI studies (and neither was observed an increase in vasopressor of fluid consumption), suggesting that potential overtreatment might not be clinically relevant if the HPI and the treatment protocol are used correctly. Despite these limitations, the positive results were confirmed in other studies. Davies et al. [14], using a more pragmatic approach, included MAP values between 65–75 mmHg and did not exclude external factors as causes of hypotension. In a retrospective study of 255 patients undergoing major surgery, the authors compared the predictive ability of HPI to other static and dynamic hemodynamic variables such as heart rate, cardiac output, ΔMAP, stroke volume, SVV, and pulse pressure, among others. Similarly to the previous study, the HPI predicted a hypotensive event 5 min before it occurred, with a sensitivity and specificity of 86% (AUC, 0.926). None of the other hemodynamic parameters showed any prediction ability for IOH [14]. 

After these initial results, four randomized controlled trials were published, comparing an HPI-guided hemodynamic treatment protocol group with a standard care control group [26,32,33,36]. The study by Wijnberge et al. [26] involved 60 patients undergoing major high-risk surgery. In the intervention group, when the value of HPI exceeded 85, a hemodynamic treatment protocol was triggered that advised physicians to act according to problems in preload, afterload, or contractility. Reduced time spent in hypotension was reported in the intervention group (2.8% vs. 10.3% of surgery time, *p* < 0.001), while the TWA of IOH was markedly higher in the control group (HPI: 0.10 mmHg vs. control: 0.44 mmHg, median difference 0.38 mmHg (*p* = 0.001)). However, the use of the HPI intraoperatively did not seem to influence postoperative hypotension occurring in the ward, as demonstrated by a substudy of this trial [42]. This latter study showed a (nonsignificant) trend in the reduction in hypotension in the HPI group but was underpowered for this question, and thus the question is not yet resolved. Adequately powered studies should be carried out in order to look into this subject further. Schneck et al. [32], in 59 patients scheduled for hip arthroplasty, compared an HPI-guided hemodynamic therapy protocol to routine care. A significant reduction in time spent in hypotension was observed in the intervention group (0% vs. 6% of the total anesthetic time, *p* < 0.001). Notably, the investigators used an HPI threshold of 80 to allow greater time for an intervention. In contrast to the previous studies, a large prospective randomized trial by Maheshwari et al. [33] failed to detect the superiority of the HPI algorithm associated with a goal-directed hemodynamic treatment protocol in 204 patients undergoing major or moderate noncardiac surgery. The investigators reported similar TWA of MAP ≤ 65 mmHg of the HPI-guided intervention group versus a usual care group (0.14 vs. 0.14 mmHg) with a median difference (95% CI) of 0 (−0.03 to 0.04), *p* = 0.757. These results were, according to the authors, attributable to noncompliance of the responsible clinicians to the treatment protocol, short warning time, and a complex treatment algorithm. Recently our group [36] published a randomized controlled trial of 99 patients undergoing moderate to major high-risk noncardiac surgery, testing the use of the HPI along with a hemodynamic treatment protocol in comparison with a standard care control group. The results confirmed previous studies [26,32], reporting a reduced TWA of IOH in the HPI intervention group (HPI: 0.16 mmHg versus control: 0.50 mmHg, median difference of −0.28, *p* = 0.0003). Of note, we also observed an increase in hypertension in the intervention group, probably as a result of overtreatment, as well as higher weight-adjusted use of phenylephrine in the intervention group.

Two retrospective studies assessed the use of HPI against a goal-directed fluid therapy (GDFT) protocol [35,39]. Both studies comprised personalized protocols for each study group and measured the TWA of IOH in both groups. Grundmann et al. [35] found that 84% of patients experienced IOH in the GDFT group, while 52% of patients were hypotensive in the HPI-guided group (*p* = 0.001) (TWA of IOH was 0.27 mmHg in the GDFT group versus 0.10 mmHg in the HPI group (*p* = 0.001)). Similarly, Solares et al. [39] reported that patients managed by following a personalized GDFT protocol experienced more IOH than HPI-guided patients (TWA 0.23 mmHg vs. 0.09 mmHg, respectively, *p* = 0.037). The length of stay in the hospital was shorter for the HPI patients, with a median difference of 2 days. A recent randomized controlled trial approached the use of the HPI in a different manner [43]. The authors used a targeted HPI value of >85 versus a MAP-guided method to manage intraoperative induced hypotension for spinal fusion surgery. They found lower intraoperative blood loss with the HPI-guided technique. Attempting to answer clinically relevant questions, Morabito et al. [40] hypothesized that treating intraoperative hypotension would result in reduced inflammatory and oxidative stress biomarkers in 40 patients undergoing elective noncardiac surgery. Confirming previous studies, they reported reduced TWA of IOH, number of hypotensive episodes, and time spent in hypotension in the intervention group. The intervention group showed lower Neuronal Specific Enolase and higher reduced glutathione compared with the control group, while other biomarkers such as neutrophil gelatinase-associated lipocalin and S100B correlated with TWA of IOH. 

#### 6.1.2. Cardiac Surgery and Intensive Care Unit

The HPI algorithm was developed from records of noncardiac surgical and intensive care patients [13], and validation of the algorithm was performed in noncardiac surgical cases [13,14]; therefore, it is of particular interest whether this prediction model can perform equally well in cardiac surgery patients.

Ranucci et al. [31] retrospectively analyzed 23 patients undergoing major vascular or cardiac surgery and found that the HPI algorithm discriminated hypotensive events with moderate sensitivity and specificity when 85 was used as the cutoff value. According to the authors, the algorithm constantly overestimated the risk of hypotension. Shin et al. [37] prospectively enrolled 37 patients undergoing elective cardiac surgery requiring CPB. In contrast to the previous study, they reported an AUC for the HPI of 0.90 5 min before the hypotensive effect with high sensitivity and specificity. Although these results may be promising and may widen the use of this algorithm in cardiac surgery, they must be validated in larger cohorts and randomized controlled trials.

Although originally, the HPI was developed and validated both in surgical and ICU patients, there is paucity in the literature for its use in the intensive care unit. One recent study involving data from 41 patients with COVID-19 admitted to the ICU found that the HPI had an optimal threshold of 90 in these patients, featuring a sensitivity of 0.91, specificity of 0.87, a positive predictive value (PPV) of 0.69, and a negative predictive value of 0.99. This validation study could provide a basis for future studies to assess whether hypotension can be reduced in ICU patients using this algorithm. 

### 6.2. Noninvasive Arterial Waveform Analysis

Four studies validating the use of the HPI with a noninvasive arterial waveform using the volume clamp method were published in the last two years. A large retrospective study involving data from 320 surgical patients evaluated the sensitivity, specificity, and PPV of the HPI based on noninvasive arterial waveform estimates [22]. The authors reported an excellent PPV at the threshold of 85 (0.83) with high sensitivity and specificity at 5 and 10 min before the hypotensive event. Likewise, Winjberge et al. [23] reported high sensitivity and specificity at the cutoff value of 85 and a PPV of 0.80 in 507 adult patients undergoing general surgery. Frassanito et al. tested the use of HPI in gynecologic oncologic patients [24] and parturients scheduled for cesarean section [25]. In the first study, they analyzed 28 patients and found that the algorithm predicted hypotensive events with a sensitivity and specificity of 0.85 (AUC = 0.95) 5 min before the event. In the 50 women who underwent cesarean section, the HPI predicted hypotensive events with a sensitivity and specificity of 83% at 3 min before the event (AUC = 0.913). It may, therefore, be formulated that HPI provides an accurate and continuous prediction of impending IOH before its occurrence in patients using a noninvasive arterial waveform analysis. These positive trials will contribute to the growth of patients who may benefit from the use of HPI.

## 7. Conclusions

The HPI is a real-time and continuous predictor of IOH that has the potential to decrease the incidence and cumulative duration of IOH. Concerns raised elsewhere [41] regarding a selection bias in the development of the model need further investigation in order to weigh whether this issue can severely flaw the algorithm performance in a clinically relevant manner. Despite this, the majority of the current literature supports its use, and the initial results are promising. Nevertheless, there is a lack of large multicenter randomized controlled trials in different clinical settings that could provide proof of its ability to produce clinically relevant outcomes in postoperative morbidity and mortality. 

## Figures and Tables

**Table 1:** Trials validating the HPI algorithm using invasive arterial waveform analysis.

Study, Year	Design	Number of Participants	Population	Primary Outcome	Results	Comments
Hatib et al., 2018 [13]	Retrospective and Prospective	554 internal validation = 350, external validation = 204	Surgical and ICU patients	Performance of the HPI algorithm using ROC analysis.	The algorithm predicted hypotension 15 min before a hypotensive event with a sensitivity and specificity of 88% and 87% (AUC, 0.95); 89% and 90% 10 min before (AUC, 0.95); 92% and 92%, 5 min before (AUC, 0.97)	Algorithm developed outside of clinical interventions that may cause hypotension; MAP values between 65 and 75 mmHg were excluded from the analyses.
Ranucci et al., 2018 [31]	Retrospective	23	Cardiac and major vascular surgery	HPI values 5 to 7 min before a hypotensive event (HPI_5–7_) were tested for discrimination and calibration, using ROC analysis	The HPI has a fair level of discrimination (AUC 0.768) and poor calibration. The cutoff value of 85% carries a sensitivity of 62.4% and a specificity of 77.7%; NPV = 97.8% and PPV = 12.6%.	The overall calibration of the HPI appears inadequate, with a constant overestimation of the risk of hypotension.
Davies et al., 2019 [14]	Retrospective	255	Major abdominal, vascular, or off-pump CABG surgery	The assessment of the diagnostic ability of the HPI or other variables in predicting hypotension, using ROC analysis.	The AUC for the prediction of hypotension, using HPI, for 5, 10, and 15 min, was 0.926, 0.895, and 0.879, respectively. The AUC for hypotension prediction using static or dynamic variables for 5, 10, and 15min was significantly lower.	The use of the HPI algorithm has a higher predictive value of an IOH event, up to 15 min before its occurrence, compared with other commonly used static hemodynamic parameters and their dynamic changes.
Schneck et al., 2019 [32]	Prospective RCT	99 (HPI = 25, control (CTRL) = 25, historical control (hCTRL) = 50)	Total hip arthroplasty under GA	Frequency ((n)/h), absolute and relative duration (% of total anesthesia time) of IOH using a threshold for HPI of 80	Significant reduction in IOH in the HPI group compared with the control groups (HPI 48%, CTRL 87.5%, hCTRL 80%; *p* < 0.001). Number of hypotensive episodes was significantly reduced in the HPI group (HPI: 0 (0–1), CTRL: 5 (2–6), hCTRL: 2 (1–3); *p* < 0.001)	Significant reduction in the incidence, as well as the absolute and relative duration of IOH events in the HPI-guided interventional group, compared with both control cohorts.
Wijnberge et al., 2020 [26]	Prospective RCT	68 (HPI = 34, non-HPI = 34)	Elective noncardiac surgery	TWA of MAP during surgery in an HPI-guided group and a standard care group.	The median TWA of IOH was 0.10 mm Hg in the HPI-guided group vs. 0.44 mmHg in the control group, for a median difference of 0.38 mmHg (*p* = 0.001)	The use of HPI, compared with standard care, resulted in less IOH.
Maheshwari et al., 2020 [33]	Prospective RCT	214 (HPI = 105, non-HPI = 109)	Moderate- or high-risk noncardiac surgery	TWA of MAP ≤ 65 mmHg in an HPI guided and a standard care group.	The median TWA of MAP < 65 mmHg was 0.14 in guided patients versus 0.14 mmHg in unguided patients: median difference (95% CI) of 0 (−0.03 to 0.04), *p* = 0.757. Post hoc guidance was associated with less hypotension when the analysis was restricted to episodes during which clinicians intervened	HPI Guided group did not reduce the TWA of MAP < 65 mmHg, probably because of inadequacies of the HPI algorithm, trial design, and clinicians’ responses to the HPI alarm.
Schenk et al., 2020 [34]	Retrospective (sub-study of [26])	54 (HPI = 28, non-HPI = 26)	Postoperative follow-up of patients that underwent elective noncardiac surgery	TWA of POH, in patients randomized in an HPI guided and a standard care group intraoperatively	POH occurred in 37/54 (68%) subjects. HPI-guided care did not reduce the TWA of POH (median difference, vs. standard of care: 0.118; 95% CI, 0–0.332; *p* = 0.112)	HPI-guided intraoperative hemodynamic care did not reduce the TWA of POH.
Grundmann et al., 2021 [35]	Retrospective observational study	100 (HPI = 50, Flotrac = 50)	Moderate- or high-risk abdominal surgery in urology, general surgery, and gynecology.	TWA of IOH; incidence and duration of IOH	The TWA of hypotension was 0.27 mmHg in the FloTrac group versus 0.1 mmHg in the HPI group (*p* = 0.001). In the FloTrac group, 42 patients (84%) experienced a hypotension, while in the HPI group 26 patients (52%) were hypotensive (*p* = 0.001).	HPI combined with personalized treatment protocols reduces hypotensive events during major abdominal surgery compared with arterial waveform analysis alone.
Tsoumpa et al., 2021 [36]	Prospective RCT	99 (HPI = 49, non-HPI = 50)	Moderate- or high-Risk noncardiac surgery	TWA of IOH in an HPI-guided group and a standard care group.	The median TWA of hypotension was 0.16 mmHg in the intervention group versus 0.50 mmHg in the control group, for a median difference of 0.28 (95% CI, 0.48 to 0.09 mmHg; *p* = 0.0003)	A significant decrease in TWA of IOH with the use of HPI. An increase in hypertensive episodes was also observed, as well as a higher weight-adjusted administration of phenylephrine, in the intervention group\
Shin et al., 2021 [37]	Prospective cohort study	37	Adult patients undergoing elective cardiac surgeries requiring CPB.	The primary outcomes were the AUC, sensitivity, and specificity of HPI predicting IOH, using ROC analysis.	The AUC, sensitivity and specificity for HPI before the hypotensive event was: 5 min: 0.90, 84%, 84%; 10 min: 0.83, 79%, 74%; and 15 min: 0.83, 79%, 74%	HPI predicted hypotensive episodes during cardiac surgeries with a high degree of sensitivity and specificity, even though HPI has been validated in noncardiac surgical patients.
van der Ven et al., 2021 [38]	Prospective cohort study	41	COVID-19 patients admitted to the ICU	Evaluation of the predictive ability of the HPI with MAP data in patients with COVID-19 admitted to the ICU for mechanical ventilation.	The HPI threshold of 80 yielded a sensitivity of 0.93 and specificity of 0.80. The HPI threshold of 85 had a sensitivity of 0.92 and specificity of 0.83. The optimal HPI threshold was 90, demonstrating a sensitivity of 0.91 and specificity of 0.87.	This validation study shows that the HPI correctly predicts hypotension in mechanically ventilated COVID-19 patients in the ICU. The HPI should also be validated on other ICU patients to translate the current results to a more heterogeneous ICU population.
Solares et al., 2022 [39]	Retrospective study	104 (HPI = 52, GDFT = 52)	Adult patients undergoing major elective or urgent noncardiac surgery with a moderate-to-high risk of bleeding.	TWA of IOH in an HPI-guided group combined with a personalized GDHT protocol and Goal-directed Fluid Therapy (GDFT) group.	The median TWA of IOH was significantly lower in the HPI than in the GDFT group (0.09 vs. 0.23; *p* = 0.037). Postoperative complications were less prevalent in the HPI patients (0.46 ± 0.98 vs. 0.88 ± 1.20), *p* = 0.035. Hospital stay was significantly shorter in HPI patients (median difference = 2 days (*p* = 0.019).	The use of HPI was associated with a significant reduction in both the severity and duration of IOH
Murabito et al., 2022 [40]	Prospective RCT	40 (HPI = 20, non-HPI = 20)	Adult patients undergoing elective major noncardiac surgery	TWA of IOH hypotension; Secondary outcomes included association with inflammatory biomarkers	TWA of IOH was lower in the HPI-guided group (0.12 mmHg in the intervention group vs. 0.37 mmHg in the control group, with a median difference of 0.25 mmHg; Neutrophil Gelatinase-Associated Lipocalin (NGAL) correlated with TWA of IOH (R = 0.32; *p* = 0.038) and S100B with a number of hypotensive episodes, absolute and relative time of hypotension, TWA of IOH (*p* < 0.001 for all).	The use of the HPI resulted in reduced intraoperative hypotension, reduced inflammatory biomarkers, and oxidative stress.

**Table 2:** Trials validating the HPI algorithm using noninvasive arterial waveform analysis.

Study, Year	Design	Number of Participants	Population	Primary Outcome	Results	Comments
Maheshwari et al., 2021 [22]	Retrospective study	320	Patients ≥ 45 yo ASA: 3–4. Moderate-to-high-risk noncardiac surgery with GA using noninvasive arterial waveform analysis	Sensitivity and specificity for predicting IOH with ROC curve analysis.	The algorithm predicted hypotension 5 min in advance, with a sensitivity and specificity of 0.86. At 10 min, the sensitivity and specificity were 0.83. At 15 min, the sensitivity and specificity were 0.75. AUC for 5, 10 and 15 min was 0.93, 0.90, 0.84, respectively.	HPI was not available at the time of data collection, so it was calculated post hoc, separately for blinded and unblinded patients. HPI works reasonably well with noninvasive arterial pressure waveform estimates.
Wijnberge et al., 2021 [23]	Observational cohort study	507	Patients undergoing general surgery, using noninvasive arterial waveform analysis	Comparison of the performance of the HPI algorithm, using noninvasive versus invasive arterial waveform analysis and assessment of the HPI alarm threshold of 85	The performance of the noninvasive HPI resulted in an AUC of 0.93, 0.91, and 0.90 at 5, 10, and 15 min, while the performance of the invasive HPI resulted in an AUC of 0.95, 0.92, and 0.91 at 5, 10, and 15 min, respectively. HPI alarm threshold of 85 showed a median time from alarm to hypotension of 2.7 min with a sensitivity of 92.7% and specificity of 87.6%. An HPI alarm threshold of 75 provided lower values but a prolonged time from prediction to actual IOH	This study demonstrated that the algorithm could be employed using continuous noninvasive arterial waveform analysis. This opens up the potential to predict and prevent hypotension in a larger patient population
Frassanito et al., 2021 [24]	Retrospective study	31	Patients undergoing gynecologic oncologic surgery, using noninvasive arterial waveform analysis	Performance of the HPI working on noninvasive blood pressure waveform to predict IOH 5, 10, and 15 min before its occurrence.	The AUC for the prediction of hypotension using HPI for 5, 10, and 15 min was, respectively, 0.93, 0.90, and 0.95. Sensitivity and specificity were both 0.85 for 15 min before the event; 0.2 and 0.83, respectively, for 10 min before the event; and 0.86 both for 5 min before the event	HPI on noninvasive arterial pressure waveform has a similar performance to HPI working on invasive arterial pressure waveform.
Frassanito et al., 2022 [25]	Retrospective study	50	Patients undergoing CS under SA, using noninvasive arterial waveform analysis	Performance of the HPI working on noninvasive blood pressure waveform to predict IOH 1, 2, and 3 min before its occurrence.	The AUC for the prediction of hypotension, using HPI, for 1, 2, and 3min, was, respectively, 1.0, 0.995, and 0.913. The AUC for the prediction of hypotension using ΔMAP, for 1, 2, and 3 min, was significantly lower.	The HPI algorithm derived from noninvasive arterial pressure waveform monitoring can predict maternal hypotension in patients undergoing CS under SA up to 3 min before the event.

Abbreviations: HPI—Hypotension Prediction Index; AUC—area under curve; NPV—negative predictive value; PPV—positive predictive value; ROC—receiver operating characteristic; CABG—coronary artery bypass graft surgery; IOH—intraoperative hypotension; GA—general anesthesia; TWA—time-weighted average; MAP—mean arterial pressure; POH—postoperative hypotension; CPB—cardiopulmonary bypass; GDFT—goal directed fluid therapy; ICU—intensive care unit; CS—cesarean section; SA—spinal anesthesia.

## Data Availability

The data presented in this study are available on request from the corresponding author.
